# Clinical characteristics and prognosis of elderly patients with colorectal cancer: Comparison between surgical resection and supportive care

**DOI:** 10.1097/MD.0000000000024609

**Published:** 2021-02-19

**Authors:** Soohwan Kim, Dong Suk Kim, Jae Seung Soh, Sang-Woo Lim, Hyun Lim, Ho Suk Kang, Jong Hyeok Kim

**Affiliations:** a Department of Internal Medicine; b Department of Colorectal Surgery, University of Hallym College of Medicine, Hallym University Sacred Heart Hospital, Anyang, Republic of Korea.

**Keywords:** colorectal cancer, elderly, prognosis, surgery

## Abstract

Elderly colorectal cancer (CRC) patients tend to avoid standard treatment, especially curative surgical resection, because of concerns about surgical complications or underlying diseases. This study is intended to compare clinical characteristics and prognosis between patients who had undergone surgical resection and received supportive care, and to evaluate the usefulness of surgical treatment in elderly patients.

A total of 114 patients aged ≥80 years who were diagnosed with CRC were analyzed retrospectively. Of these patients, 73 patients underwent surgical resection for malignancy and 41 patients received supportive care. Clinicopathological factors and overall survival (OS) rates were compared.

The **s**urgical resection group had better Eastern Cooperative Oncology Group performance status, American Society of Anesthesiologists (ASA) physical status, and a lower stage than did the supportive-care group. The 3-year OS rate of the surgical group was significantly higher than that of the supportive-care group (60.7% vs 9.1%, *P* < .001). In extremely elderly patients (age ≥85 years), the surgical group showed a better 3-year OS rate than did the supportive-care group (73.9% vs 6.3%, *P* < .001), although Eastern Cooperative Oncology Group performance status and ASA physical status were not different. The post-operative mortality rate was 2.7%. In the analysis of risk factors related to survival, surgical resection was a good prognostic factor.

Surgical treatment in elderly CRC patients showed a survival benefit, even in the extremely elderly patients. Surgical resection for CRC in elderly patients can be considered to improve survival.

## 1 Introduction

Colorectal cancer (CRC) is the third most commonly diagnosed malignancy and second leading cause of cancer death in the world. Elderly people have a higher incidence and mortality rate of CRC than do younger people. With the aging population, around 20% of patients diagnosed with CRC are more than 80 years old in the United States.^[1,2]^

Elderly patients tend to avoid standard treatment, including curative resection of malignancy, because of the concerns about surgical complications and comorbidities, and go through supportive or palliative care rather than active treatment for cancer. As a result, a large proportion of elderly patients with CRC may receive sub-standard treatment.^[3]^ However, studies found that survival of elderly patients who receive anticancer treatment including surgery or chemotherapy could be meaningfully improved more than that of those who received only supportive care.^[4,5]^

CRC presents with many complications, including obstruction, bleeding, and perforation at the diagnosis, and surgery plays a major role in treating CRC. Recent advances in technology, skills, and studies have made it possible for elderly patients to undergo surgical treatment relatively safely. It has been found that surgical resection of CRC can be tolerated well, especially if done laparoscopically in elderly patients.^[6,7]^ Therefore, decisions about surgical resection for elderly CRC patients should consider various conditions of the patients besides age.

Our aim in this study is to compare clinical characteristics and prognosis between patients aged ≥80 years who had undergone surgical resection and received supportive care, and to evaluate the usefulness of surgical treatment in elderly patients. We also did prognostic analysis related to survival in elderly patients with CRC.

## 2 Materials and methods

### 2.1 Study population

Between March 2007 and November 2017, a total of 125 patients aged ≥80 years old were diagnosed with colorectal adenocarcinoma at the Hallym University Sacred Heart Hospital in Anyang, Korea. We reviewed these patients retrospectively and classified them according to treatment modalities and age. Of the 125 patients, 11 were excluded for resecting with endoscopic submucosal dissection or endoscopic mucosal resection (4 patients), transfer to another hospital after the diagnosis of cancer (4 patients), and no available clinical information, including treatment and outcome for analysis (3 patients). A total of 114 patients were included in our study. Of these patients, 44 were more than 85 years old, and they were defined as the extremely elderly group. Clinical, pathological, and outcome data were collected by reviewing patient medical records and by interviewing patients by phone. The local ethics committee at Hallym Sacred Heart Medical Center approved the use of clinical data for this study (IRB 2018–09–011).

### 2.2 Clinicopathological data

The following patients characteristics were analyzed: age, sex, comorbidity disease, body mass index (BMI), hemoglobin and carcinoembryonic antigen (CEA) level, performance status (Eastern Cooperative Oncology Group [ECOG]) at initial diagnosis,^[8]^ American Society of Anesthesiologists (ASA) classification,^[9]^ tumor location, clinical stage, lymph-node metastasis, tumor size, histological type, perforation, therapeutic modalities, and treatment outcomes. BMI was calculated as weight (kg) divided by height squared (m^2^) at initial diagnosis. Comorbidity diseases were defined as follows:

(1) cardiovascular disease included patients with coronary artery disease (myocardial infarction, angina) and heart failure;
(2) cerebral disease included patients with a history of a cerebrovascular accident and transient ischemic attacks;
(3) pulmonary disease included patients with asthma, chronic bronchitis, emphysema, and other chronic lung disease;
(4) renal disease included chronic kidney disease.

Tumor location was divided into right side of the colon (including cecum, ascending colon, and transverse colon) and left side of the colon (including descending colon, sigmoid colon, and rectum). Preoperative clinical staging was established by enhanced computed tomography (CT). The long diameter of an enlarged lymph node greater than 0.8 cm on CT image was defined as lymph-node metastasis. Distant metastasis was defined as the cancer spreading to organs or lymph nodes far away from the colon. The differentiation of tumor was evaluated according to the percentage portion of the tumor exhibiting glandular structures between differentiated (well and moderately differentiated adenocarcinoma) and undifferentiated (poorly differentiated adenocarcinoma, signet-ring cell carcinoma, and mucinous adenocarcinoma). Post-operative mortality was defined as being within 30 days of surgery.

### 2.3 Statistical analyses

Baseline characteristics and clinical outcomes were compared between the surgery group and the supportive-care group. Continuous variables were compared by Student *t* tests, and categorical variables were compared with chi-square tests or Fisher's exact tests. All *P* < .05 were considered to be statistically significant. We did a survival analysis using Kaplan–Meier curves and compared these using the log-rank test by a Cox regression analysis. The overall survival (OS) was defined as the time from the date of diagnosis to the date of death. We used the SPSS software (version 22.0; SPSS, Chicago, IL) for all statistical analyses.

## 3 Results

### 3.1 Baseline characteristics of the patients according to the treatment modality

Of the 114 patients with CRC, 73 had surgical resection and/or postoperative chemotherapy for malignancy (surgery group) and 41 had supportive care without surgical resection, chemotherapy, or radiation therapy (supportive-care group). Among the 41 patients in the supportive-care group, 6 patients had bypass surgery without resection of cancer lesions. Baseline characteristics of the patients according to the treatment modality are shown in Table 1. The surgery group was younger than the supportive-care group (*P* = .028). The supportive-care group showed higher ECOG performance status, ASA grade, clinical stage, and more lymph node metastasis (*P* = .009, .001, .001, and .002, respectively).

**Table 1:** Baseline characteristics of the patients according to the treatment modality.

	Surgery (n = 73)	Supportive-care (n = 41)	*P* value
Age, mean, years (range)	83.6 (80–94)	85.0 (80–94)	**.028**
Male sex, no. (%)	29 (39.7)	14 (34.1)	.688
Comorbidity			
HTN, no. (%)	47 (64.4)	23 (56.1)	.426
DM, no. (%)	20 (27.4)	9 (22.0)	.655
Cardiovascular disease, no. (%)	9 (12.3)	9 (22.0)	.191
Cerebral disease, no. (%)	18 (24.7)	11 (26.8)	.825
Pulmonary disease, no. (%)	4 (5.5)	6 (14.6)	.164
Renal disease, no. (%)	3 (4.1)	3 (7.3)	.665
Other malignancy, no. (%)	5 (6.8)	4 (9.8)	.720
BMI, mean, kg/m^2^ (range)	22.8 (15.9–30.2)	21.6 (14.2–29.7)	.065
Hemoglobin, mean, g/dL (range)	10.8 (5.4–17.3)	10.2 (4.0–14.8)	.225
CEA ≥5 ng/mL, no. (%)	29 (39.7)	24 (58.5)	.078
Performance (ECOG) status			**.009**
0–2, no. (%)	51 (69.9)	18 (43.9)	
3–4, no. (%)	22 (30.1)	23 (56.1)	
ASA grade			**<.001**
I, II, no. (%)	38 (52.1)	5 (12.2)	
III, IV, no. (%)	35 (47.9)	36 (87.8)	
Location of tumor			.560
Right colon, no. (%)	36 (49.3)	23 (56.1)	
Left colon, no. (%)	47 (50.7)	18 (43.9)	
Clinical stage			**<.001**
I, no. (%)	9 (12.3)	3 (7.3)	
II, no. (%)	33 (45.2)	6 (14.6)	
III, no. (%)	23 (31.5)	10 (24.4)	
IV, no. (%)	8 (11.0)	22 (53.7)	
Lymph node metastasis, no. (%)	30 (41.1)	30 (73.2)	**.002**
Tumor size, mean, cm (range)	4.7 (1.0–12.7)	5.3 (2.0–12.7)	.195
Histologic type			.087
Differentiated	68 (93.2)	26 (81.3)	
Undifferentiated	5 (6.8)	6 (18.8)	
Perforation, no. (%)	13 (17.8)	3 (7.3)	.163

### 3.2 Prognostic analyses for overall survival in the patients

**Figure 1:**
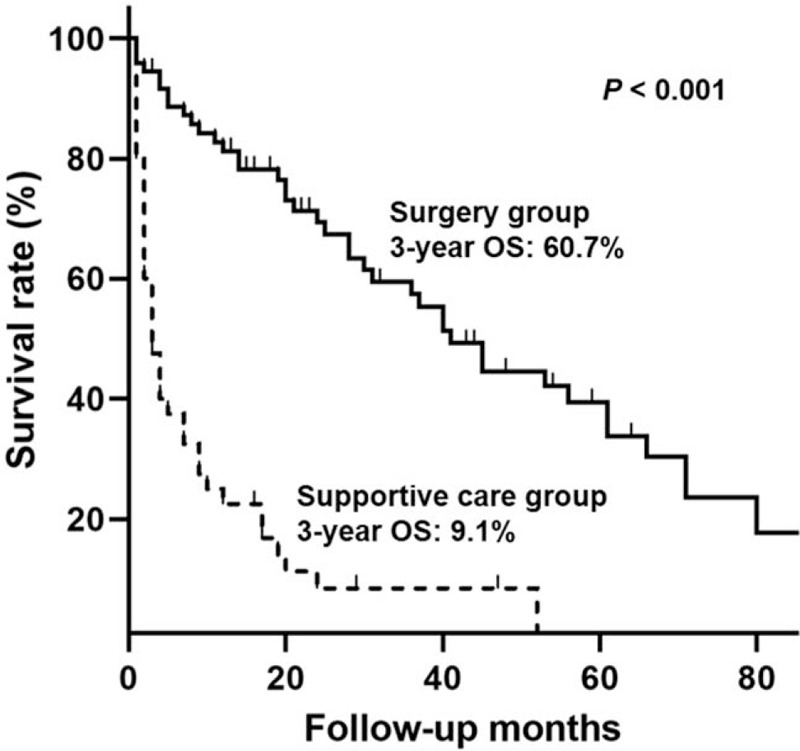
The overall survival curves of the patients according to the treatment modality using Kaplan-Meier estimation.

Forty-two patients died in the surgery group (57.5%) compared to 37 patients (90.2%) in the supportive-care group during the follow-up period. The mean follow-up period was 28 months, ranging from 1 to 121 months. Figure 1 shows the OS curves of the patients according to the treatment modality using Kaplan-Meier estimation. The OS rate of the surgery group was significantly higher than that of the supportive-care group (*P* < .001). The 3-year OS rates of the surgery and supportive-care groups were 60.7% and 9.1%, respectively. The post-operative mortality rate of the surgery group was 2.7% (2/73).

Prognostic factors related to the OS were investigated by univariate and multivariate analysis (Table 2). CEA ≥5 ng/mL (*P* = .012), ASA grade (*P* = .017), lymph-node metastasis (*P* = .001), tumor size (*P* = .026), and surgical treatment modality (*P* = .001) had a statistically significant association with overall survival by univariate analysis. However, only the surgical treatment modality was associated with better OS in multivariate analysis. (hazard ratio [HR] = 0.235; *P* = .019).

**Table 2:** Univariate and multivariate analyses of risk factors for overall survival.

	Univariate analysis			Multivariate analysis		
	HR	95% CI	*P* value	HR	95% CI	*P* value
Age, 1-year increase	1.050	0.926–1.190	.449			
Male sex	0.808	0.352–1.855	.615			
Comorbidity						
HTN	0.435	0.181–1047	.063			
DM	0.979	0.393–2.438	.964			
Cardiovascular disease	1.669	0.507–5.491	.399			
Cerebral disease	1.222	0.480–3.108	.674			
Pulmonary disease	1.859	0.374–9.241	.448			
Renal disease	2.297	0.258–20.426	.456			
Other malignancy	3.831	0.460–31.874	.214			
BMI ≥25 kg/m^2^	0.979	0.393–2.438	.964			
Hemoglobin ≥8 g/dL	0.632	0.120–3.329	.632			
CEA ≥5 ng/mL	2.986	1.268–7.033	**.012**	2.155	0.840–5.534	.110
Performance (ECOG) status						
0–2	1					
3–4	1.989	0.844–4.687	.116			
ASA grade						
I, II	1					
III, IV	2.721	1.199–6.178	**.017**	1.260	0.496–3.203	.627
Location of tumor						
Right colon	1					
Left colon	1.158	0.521–2.572	.719			
Lymph node metastasis	4.310	1.816–10.230	**.001**	2.439	0.900–6.613	.080
Tumor size, 1 cm increase	1.304	1.032–1.647	**.026**	1.137	0.877–1.474	.333
Histologic type						
Differentiated	1					
Undifferentiated	1.312	0.325–5.294	.703			
Perforation	0.700	0.233–2.107	.526			
Treatment modality						
Supportive-care	1					
Surgery	0.146	0.047–0.454	**.001**	0.235	0.070–0.789	**.019**

### 3.3 Baseline characteristics of the extremely elderly group according to the treatment modality

Of 44 patients aged ≥85 years, 23 patients had surgical resection and/or chemotherapy of malignancy and 21 patients received supportive care. Table 3 shows the baseline characteristics of the extremely elderly group according to the treatment modality. There were no significant differences between the surgery group and supportive-care group including CEA level, ECOG performance status, ASA grade, and lymph-node metastasis. The surgery group showed a lower clinical stage than did the supportive-care group (*P* < .001).

**Table 3:** Baseline characteristics of the extremely elderly group according to the treatment modality.

	Surgery (n = 23)	Supportive-care (n = 21)	*P* value
Age, mean, years (range)	87.5 (85–94)	87.7 (85–94)	.791
Male sex, no. (%)	7 (30.4)	6 (28.6)	1.000
Comorbidity			
HTN, no. (%)	19 (82.6)	11 (52.4)	.052
DM, no. (%)	5 (21.7)	3 (14.3)	.701
Cardiovascular disease, no. (%)	2 (8.7)	5 (23.8)	.232
Cerebral disease, no. (%)	5 (21.7)	2 (9.5)	.416
Pulmonary disease, no. (%)	2 (8.7)	2 (9.5)	1.000
Renal disease, no. (%)	1 (4.3)	1 (4.8)	1.000
Other malignancy, no. (%)	3 (13.0)	1 (4.8)	.609
BMI, mean, kg/m^2^ (range)	23.1 (16.6–30.2)	21.5 (15.2–29.3)	.178
Hemoglobin, mean, g/dL (range)	10.6 (6.1–15.1)	9.9 (6.1–14.8)	.420
CEA ≥5 ng/mL, no. (%)	9 (39.1)	12 (57.1)	.365
Performance (ECOG) status			1.000
0–2, no. (%)	13 (56.5)	11 (52.4)	
3–4, no. (%)	10 (43.5)	10 (47.6)	
ASA grade			.462
I,II, no. (%)	6 (26.1)	3 (14.3)	
III, IV, no. (%)	17 (73.9)	18 (85.7)	
Location of tumor			.533
Right colon, no. (%)	16 (69.6)	12 (57.1)	
Left colon, no. (%)	7 (30.4)	9 (42.9)	
Clinical stage			**<.001**
I, no. (%)	2 (8.4)	1 (4.8)	
II, no. (%)	12 (52.2)	4 (19.0)	
III, no. (%)	7 (30.4)	2 (9.5)	
IV, no. (%)	2 (8.7)	14 (66.7)	
Lymph node metastasis, no. (%)	9 (39.1)	14 (66.7)	.080
Tumor size, mean, cm (range)	4.5 (2.0–8.0)	5.1 (2.5–12.7)	.321
Perforation, no. (%)	4 (17.4)	1 (4.8)	.348

### 3.4 Prognostic analyses for overall survival in the extremely elderly group

**Figure 2:**
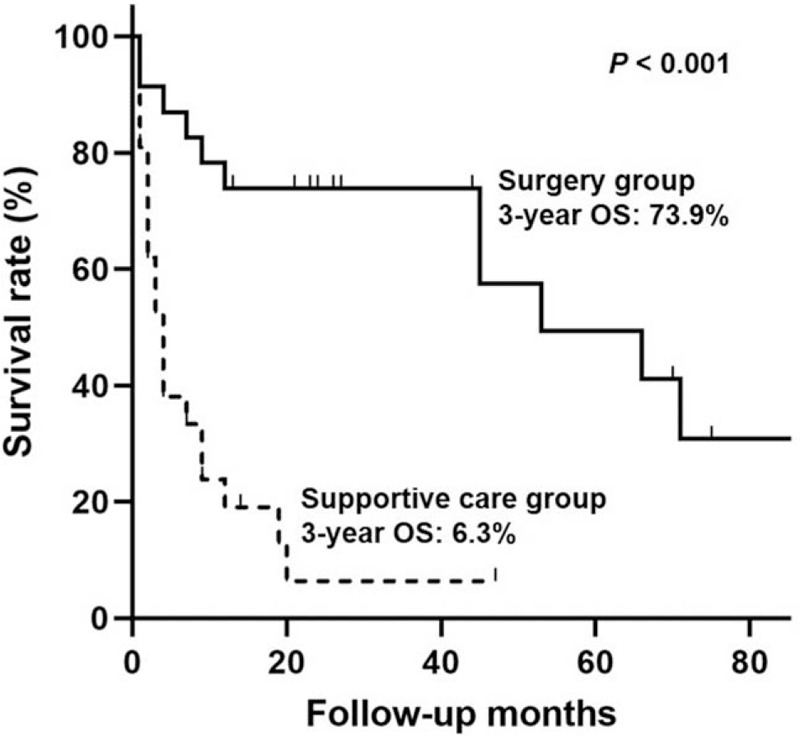
The overall survival curves of the extremely elderly group according to the treatment modality using Kaplan-Meier estimation.

During the mean follow-up of 23 months (range, 1–96 months), 11 patients expired in the surgery group (47.8%) and 19 patients (90.5%) died in the supportive-care group. The OS curves showed that the surgery group had a higher OS rate than did the supportive-care group (Fig. 2; *P* < .001). The 3-year OS rate of the surgery group was 73.9% and that of the supportive-care group was 6.3%. The post-operative mortality rate was 4.3% (1/23).

The risk factors associated with OS in the extremely elderly group are indicated in Table 4. In univariate analysis, CEA ≥5 ng/mL (*P* = .023), lymph-node metastasis (*P* = .037), and surgical treatment modality (*P* = .006) showed a statistical significance. In multivariate analysis, surgical treatment modality was associated with better overall survival (HR = 0.083; *P* = .009) and CEA ≥5 ng/mL was inversely related to good overall survival (HR = 5.635; *P* = .039).

**Table 4:** Univariate and multivariate analyses of risk factors for overall survival in the extremely elderly group.

	Univariate analysis			Multivariate analysis		
	HR	95% CI	*P* value	HR	95% CI	*P* value
Age, 1-year increase	1.317	0.927–1.871	.124			
Male sex	1.833	0.415–8.096	.424			
Comorbidity						
HTN	0.614	0.211–1.785	.370			
DM	0.385	0.080–1.841	.232			
Cardiovascular disease	1.200	0.203–7.105	.841			
cCerebral disease	0.278	0.053–1.466	.131			
Pulmonary disease	1.444	0.137–15.266	.760			
Renal disease	0.448	0.026–7.734	.581			
Other malignancy	1.444	0.137–15.266	.760			
BMI ≥25 kg/m^2^	0.450	0.110–1.848	.268			
Hemoglobin ≥8 g/dL	0.389	0.072–2.104	.273			
CEA ≥5 ng/mL	5.500	1.264–23.940	**.023**	5.635	1.089–29.166	**.039**
Performance (ECOG) status						
0–2	1					
3–4	1.167	0.325–4.190	.813			
ASA grade						
I, II	1					
III, IV	2.000	0.444–9.013	.367			
Location of tumor						
Right colon	1					
Left colon	1.667	0.423–6.562	.465			
Lymph node metastasis	4.318	1.090–17.112	**.037**	0.837	0.572–1.227	.363
Tumor size, 1 cm increase	0.973	0.690–1.373	.876			
Histologic type						
Differentiated	1					
Undifferentiated	2.182	0.218–21.793	.506			
Perforation	0.262	0.038–1.787	.171			
Treatment modality						
Supportive-care	1					
Surgery	0.096	0.018–0.513	**.006**	0.083	0.013–0.536	**.009**

### 3.5 Baseline characteristics of the localized group according to the treatment modality

We evaluated the patients diagnosed with stage I to III by preoperative clinical staging, which was defined as the localized group. Of 84 patients without distant metastasis, 65 patients had surgical resection and 19 patients received supportive care. Baseline characteristics of the localized group according to the treatment modality are shown in Table 5. The surgery group had more pulmonary disease than the supportive-care group (*P* = .025). The supportive-care group showed higher ECOG performance status (*P* = .005) and ASA grade (*P* = .035). However, clinical stage and lymph node metastasis were not different between 2 groups.

**Table 5:** Baseline characteristics of the localized group according to the treatment modality.

	Surgery (n = 65)	Supportive care (n = 19)	*P* value
Age, mean, years (range)	83.6 (80–94)	84.2 (80–94)	.520
Male sex, no. (%)	25 (38.5)	5 (26.3)	.420
Comorbidity			
HTN, no. (%)	44 (67.7)	11 (57.9)	.428
DM, no. (%)	18 (27.7)	4 (21.1)	.768
Cardiovascular disease, no. (%)	9 (13.8)	4 (21.1)	.478
Cerebral disease, no. (%)	15 (23.1)	7 (36.8)	.247
Pulmonary disease, no. (%)	4 (6.2)	5 (26.3)	**.025**
Renal disease, no. (%)	3 (4.6)	1 (5.3)	1.000
Other malignancy, no. (%)	4 (6.2)	1 (5.3)	1.000
BMI, mean, kg/m^2^ (range)	22.8 (15.9–30.2)	21.7 (14.2–29.7)	.267
Hemoglobin, mean, g/dL (range)	10.8 (5.4–17.3)	10.2 (4.0–14.0)	.441
CEA ≥5 ng/mL, no. (%)	24 (36.9)	10 (52.6)	.289
Performance (EGOG) status			**.005**
0–2, no. (%)	48 (73.8)	7 (36.8)	
3–4, no. (%)	17 (26.2)	12 (63.2)	
ASA grade			**.035**
I,II, no. (%)	37 (56.9)	5 (26.3)	
III, IV, no. (%)	28 (43.1)	14 (73.7)	
Location of tumor			.613
Right colon, no. (%)	33 (50.8)	11 (57.9)	
Left colon, no. (%)	32 (49.2)	8 (42.1)	
Clinical stage			.396
I, no. (%)	9 (13.8)	3 (15.8)	
II, no. (%)	33 (50.8)	6 (31.8)	
III, no. (%)	23 (35.4)	10 (52.6)	
Lymph node metastasis, no. (%)	23 (35.4)	10 (52.6)	.193
Tumor size, mean, cm (range)	4.6 (1.0–10.0)	4.9 (2.5–10.0)	.603
Perforation, no. (%)	11 (16.9)	2 (10.5)	.723

### 3.6 Prognostic analyses for overall survival in the localized group

**Figure 3:**
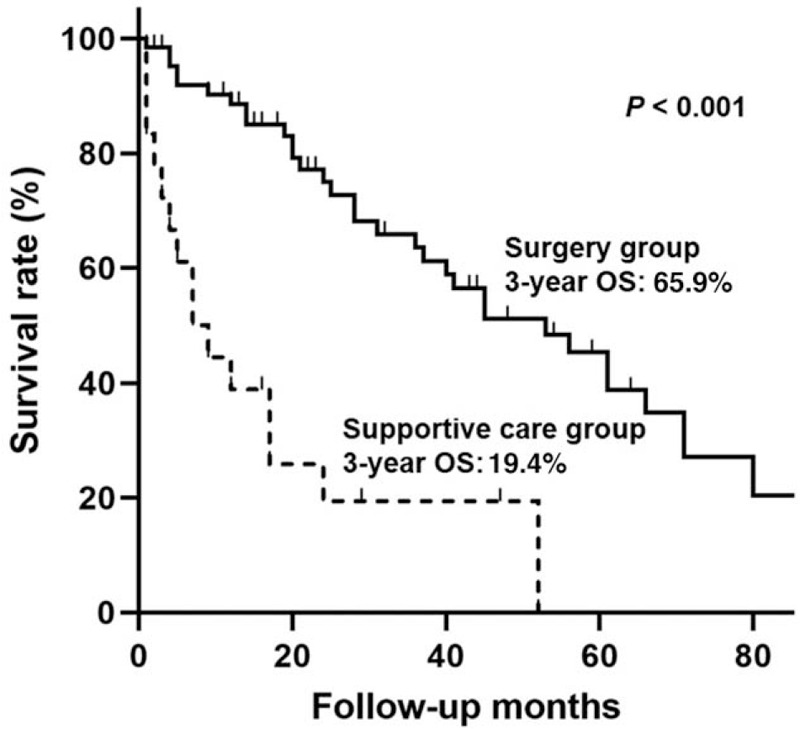
The overall survival curves of the localized group according to the treatment modality using Kaplan-Meier estimation.

During the mean follow-up of 30 months (range, 1–121 months), 34 patients expired in the surgery group (52.3%) and 15 patients (78.9%) died in the supportive-care group. The surgery group had a significantly higher OS rate than the supportive-care group (Fig. 3; *P* < .001). The 3-year OS rate of the surgery group was 65.9% and that of the supportive-care group was 19.4%.

Table 6 shows the risk factors associated with OS in the localized group. In univariate analysis, lymph-node metastasis (*P* = .034) and surgical treatment modality (*P* = .015) showed a statistical significance. In multivariate analysis, surgical treatment modality was only associated with better OS (HR = 0.224; *P* = .030).

**Table 6:** Univariate and multivariate analyses of risk factors for overall survival in the localized group.

	Univariate analysis			Multivariate analysis		
	HR	95% CI	*P* value	HR	95% CI	*P* value
Age, 1-year increase	0.994	0.868–1.139	.936			
Male sex	0.899	0.362–2.228	.817			
Comorbidity						
HTN	0.665	0.267–1.656	.380			
DM	1.043	0.388–2.803	.933			
Cardiovascular disease	1.744	0.491–6.195	.390			
Cerebral disease	1.350	0.495–3.682	.558			
Pulmonary disease	2.750	0.535–14.124	.226			
Renal disease	2.217	0.221–22.254	.499			
Other malignancy	3.022	0.323–28.278	.332			
BMI ≥25 kg/m^2^	1.275	0.482–3.367	.625			
Hemoglobin ≥8 g/dL	0.503	0.144–1.760	.282			
CEA ≥5 ng/mL	2.400	0.954–6.039	.063			
Performance (EGOG) status						
0–2	1					
3–4	1.583	0.624–4.020	.334			
ASA grade						
I, II	1					
III, IV	1.342	0.562–3.204	.507			
Location of tumor						
Right colon	1					
Left colon	1.140	0.478–2.720	.768			
Lymph node metastasis	2.773	1.081–7.117	**.034**	2.348	0.883–6.242	.087
Tumor size, 1 cm increase	1.238	0.965–1.588	.092			
Histologic type						
Differentiated	1					
Undifferentiated	1.260	0.280–5.679	.763			
Perforation	0.806	0.245–2.644	.721			
Treatment modality						
Supportive care	1					
Surgery	0.193	0.051–0.728	**.015**	0.224	0.058–0.864	**.030**

### 3.7 Pathology of the surgery group

Table 7 shows the operation methods and pathology of the surgery group. Thirty-five patients received anterior resection or the Hartmann operation and 30 patients had a right hemicolectomy. One patient received a right hemicolectomy and anterior resection for treating two malignant lesions simultaneously. Pathological stage III was most common (74.0%), and lymph-node invasion was detected in 31 patients (42.5%). Of eight patients with metastasis (4 patients with liver metastasis, 2 patients with liver and lung metastasis, and 2 patients with paraaortic lymph-node metastasis), six patients were given a palliative surgical resection for obstruction. The other two patients with liver metastasis were given bowel resection with hepatectomy for curative surgical treatments. The mean size of tumors was 5.2 cm, and poorly differentiated malignancy was 6.8%. Lymphatic, vascular, and perineural invasions of the pathological specimens were 37.0%, 17.8%, and 19.2%, respectively. Postoperative chemotherapy was done in 15 patients (20.5%), and recurrence was detected in 12 patients during the follow-up periods.

**Table 7:** Pathology of the surgery group.

	Surgery group (n = 73)
Operation method	
Anterior resection or Hartmann, no. (%)	35 (47.9)
Right hemicolectomy, no. (%)	30 (41.1)
Left hemicolectomy, no. (%)	5 (6.8)
Right hemicolectomy + anterior resection, no. (%)	1 (1.4)
Transverse colectomy, no. (%)	1 (1.4)
Cecectomy, no. (%)	1 (1.4)
pT stage	
I, no. (%)	4 (5.5)
II, no. (%)	10 (13.7)
III, no. (%)	54 (74.0)
IV, no. (%)	5 (6.8)
pN stage	
0, no. (%)	42 (57.5)
I, no. (%)	22 (30.1)
II, no. (%)	9 (12.3)
Surgical method in patients with stage IV	
Anterior resection or Hartmann, palliative, no. (%)	4 (50.0)
Right hemicolectomy, palliative, no. (%)	2 (25.0)
Anterior resection + hepatectomy, no. (%)	2 (25.0)
Tumor size, mean, cm (range)	5.2 (1.0–17.0)
Histologic type	
Well-differentiated, no. (%)	22 (30.1)
Moderately differentiated, no. (%)	46 (63.1)
Poorly differentiated, no. (%)	5 (6.8)
Lymphatic invasion, no. (%)	27 (37.0)
Vascular invasion, no. (%)	13 (17.8)
Perineural invasion, no. (%)	14 (19.2)
Postoperative chemotherapy, no. (%)	15 (20.5)
Recurrence, no. (%)	12 (16.4)

## 4 Discussion

The number of elderly patients with CRC is expected to rise, especially in developed countries with an aging society, and it is strongly correlated with affluent lifestyles, such as increased meat consumption, and obesity.^[2]^ Because the CRC incidence rate is much higher in the elderly population, a proper treatment modality for CRC patients of advanced age is critical in an aging society. Poor performance status, higher incidence of comorbidities, and lower life expectancy of elderly patients lead them to decline sufficient treatment, such as radical surgery, and can lead to less optimal treatment. However, surgical treatment is still the first choice even in CRC patients of advanced age. Our results showed that patients aged ≥80 years benefit in survival from surgical resection. In addition, extremely elderly CRC patients aged ≥85 years showed a survival benefit in the surgery group, despite having performance and ASA status similar to that of the supportive-care group.

Published data on the treatment of elderly patients with CRC are limited. Several studies showed favorable results for surgery in elderly CRC patients. Surgical resection of CRC can lead to a complete cure, especially in localized disease and better survival even in selected metastatic diseases.^[5,10]^ Tolerability of surgery by elderly patients aged ≥80 years has been investigated in several studies and showed favorable outcomes in terms of postoperative mortality as compared with that of younger patients.^[11–13]^ When “elderly” was defined as ≥75 years, the cancer-specific survival rate of elderly CRC patients receiving surgical treatment was not significantly different from that of younger patients.^[14–16]^ However, a few studies showed that emergency operations were more common in elderly CRC patients; therefore, they had more complications and post-operative mortality than did younger patients.^[14,17,18]^ Because of a higher risk of treatment-related complications, elderly CRC patients have shown a poorer prognosis than have young CRC patients.^[19]^

A previous study from our hospital compared the survival rates of the surgery group with those of the supportive-care group in elderly patents aged ≥80 years with advanced gastric cancer.^[20]^ This study showed that patients aged 80 to 85 years could expect a better prognosis with surgical resection, and surgical resection in extremely elderly patients aged ≥85 years also tended to be associated good prognosis, although there was no statistical significance. In this study, we found that the surgical treatment modality in CRC patients was significantly associated with better OS in both the elderly and the extremely elderly groups. The post-operative mortality rates for gastric cancer and CRC in our institution were 6.1% (3/49) and 2.7% (2/73), respectively; that showed relatively low morbidity after surgical treatment. With these results, cancer surgery could be considered positively for patients aged ≥80 years.

One important prognostic factor in deciding on operability for elderly patients with CRC was performance status. The surgery group had more patients with good performance status than did the supportive-care group (ECOG performance status 0–2; 69.9% vs 54.9%, *P* = .009). However, performance status was not a significant prognostic factor for OS in our study. Performance status was subjective and might be different according to physicians. In addition, patients with good performance status often refused surgical resection considering their advanced age and comorbidities by themselves or their families. In the supportive-care group, patients with good performance status had longer mean survival than did patients with poor performance status, although it did not reach statistical significance (12.9 months vs 5.7 months, *P* = .078). Another factor in deciding on operability was clinical stage at the diagnosis of CRC. More patients were clinical stage IV in the supportive-care group than in the surgery group (53.7% vs 11.0%, *P* < .001). Among eight patients with metastatic disease in the surgery group, two had radical surgery, including hepatectomy, and the other 6 patients had palliative surgery. However, the mean survival durations of surgery patients with metastatic disease were not different from those of the supportive-care group (12.5 months vs 8.9 months, *P* = .450). Although surgery was associated with better OS, elderly patients should decide on the treatment modality considering their own conditions and disease status.

Except for performance status and clinical stage, treatment of elderly patients with CRC was decided by considering various factors, such as comorbidities, CEA level, lymph-node involvement, tumor location, tumor size, and histologic differentiation. In this study, CEA ≥5 ng/mL, ASA grade, lymph-node metastasis, and tumor size were risk factors correlated with OS in the univariate analysis. Detailed examination of the various clinicopathological factors presented in the diagnosis is important for deciding on the treatment modality in elderly patients with CRC. A multidisciplinary team approach has been increasingly used, and a comprehensive geriatric assessment can be helpful in identifying patients who can tolerate surgery without significant complications.^[21,22]^ Only after considering risks and benefits of surgery should surgical resection be done in elderly CRC patients.

Our study had several limitations. First, the analysis was retrospective and was a small study conducted in a single center. There may be unrecognized or unmeasured biases. Second, background factors including performance status, ASA grade, and clinical stage were different between the two groups, which might have influenced the prognosis in the analysis, and the supportive-care group cannot be clearly considered to be a control group for the surgery group. Third, disease-specific survival rate was not shown in this study. Because all patients were advanced in age, investigating specific causes of death was impossible. Lastly, means and quality of supportive care were varied in our study. Fluid therapy with electrolyte, active pain control, and endoscopic metal-stent insertion for cancer obstruction were selectively conducted in the supportive-care group. Intensive patient care might have an effect on OS. Detailed analysis according to various ways of supportive care was needed for comparing the prognosis with surgery group.

In conclusion, our results showed that elderly patients with CRC had a survival benefit from surgical resection. In the extremely elderly patients aged ≥85 years, surgery was associated with a better OS rate than the supportive care had, although the two groups had a similar performance status and ASA grade. Surgical resection for CRC in elderly patients can be done to improve their survival rate after considering various clinicopathologic factors of patients.

## Author contributions

**Conceptualization:** Jae Seung Soh, Soohwan Kim, Sang-Woo Lim, Hyun Lim.

**Data curation:** Jae Seung Soh, Dong Suk Kim.

**Formal analysis:** Jae Seung Soh.

**Investigation:** Jae Seung Soh.

**Methodology:** Jae Seung Soh.

**Supervision:** Sang-Woo Lim, Hyun Lim, Ho Suk Kang.

**Writing – original draft:** Jae Seung Soh, Soohwan Kim.

**Writing – review & editing:** Jae Seung Soh, Sang-Woo Lim, Hyun Lim, Ho Suk Kang, Jong Hyeok Kim.
